# Using Multiple Decomposition Methods and Cluster Analysis to Find and Categorize Typical Patterns of EEG Activity in Motor Imagery Brain–Computer Interface Experiments

**DOI:** 10.3389/frobt.2020.00088

**Published:** 2020-07-30

**Authors:** Alexander Frolov, Pavel Bobrov, Elena Biryukova, Mikhail Isaev, Yaroslav Kerechanin, Dmitry Bobrov, Alexander Lekin

**Affiliations:** ^1^Research Institute of Translational Medicine, Pirogov Russian National Research Medical University, Moscow, Russia; ^2^Institute of Higher Nervous Activity and Neurophysiology, Russian Academy of Science, Moscow, Russia

**Keywords:** brain–computer interface, motor imagery, blind source separation, independent component analysis, common spatial patterns, cluster analysis, EEG pattern extraction

## Abstract

In this study, the sources of EEG activity in motor imagery brain–computer interface (BCI) control experiments were investigated. Sixteen linear decomposition methods for EEG source separation were compared according to different criteria. The criteria were mutual information reduction between the source activities and physiological plausibility. The latter was tested by estimating the dipolarity of the source topographic maps, i.e., the accuracy of approximating the map by potential distribution from a single current dipole, as well as by the specificity of the source activity for different motor imagery tasks. The decomposition methods were also compared according to the number of shared components found. The results indicate that most of the dipolar components are found by the Independent Component Analysis Methods AMICA and PWCICA, which also provided the highest information reduction. These two methods also found the most task-specific EEG patterns of the blind source separation algorithms used. They are outperformed only by non-blind Common Spatial Pattern methods in terms of pattern specificity. The components found by all of the methods were clustered using the Attractor Neural Network with Increasing Activity. The results of the cluster analysis revealed the most frequent patterns of electrical activity occurring in the experiments. The patterns reflect blinking, eye movements, sensorimotor rhythm suppression during the motor imagery, and activations in the precuneus, supplementary motor area, and premotor areas of both hemispheres. Overall, multi-method decomposition with subsequent clustering and task-specificity estimation is a viable and informative procedure for processing the recordings of electrophysiological experiments.

## Introduction

A brain–computer interface (BCI) is a system for controlling a device using registered brain activity. To control the device, the BCI operator is usually required to perform different mental tasks and does not need to use any muscles. In practice, BCI systems are intended to create new means of communication between the operator and his/her environment, which is essential for the paralyzed. Recently, BCI technology has been used not to create a new communication channel but rather to enhance the resting one lost due to stroke or trauma. BCI rehabilitation applications are a growing field, with more clinical data reported every year (Ang et al., 2011, 2015; Ramos-Murguialday et al., 2013; Ono et al., 2014; Frolov et al., 2017b; Mane et al., 2019; Bai et al., 2020). Aside from in the clinic, these systems can be used in fundamental research. The feedback provided by BCI system operation makes its user highly concentrated on performing the tasks required to control it. The user has to produce stable and repeatable patterns of the brain activity recorded, greatly facilitating the investigation of the task-specific patterns of brain activity (Frolov et al., 2012).

Although there are numerous BCI classifiers, i.e., algorithms for brain activity pattern classification, these algorithms typically do not provide pattern features that have clear physiological interpretation. In this work, we have decided to investigate the EEG recordings of our BCI experiments with different source separation methods. Methods such as Principal and Independent Component Analysis (ICA, Hyvärinen et al., 2004), as well as other Blind Source Separation (BSS, Cichocki and Amari, 2002) techniques and non-blind Common Spatial Patterns (CSP, Bashashati et al., 2007), are widely used in EEG research both in the BCI and non-BCI fields. As will be described later, all of the methods considered decompose the signal into the sum of sources, or components, each characterized by its topographic map of weights and temporal activity.

Our experiments are mainly focused on the investigation of the performance of different motor imagery tasks while controlling the BCI (Frolov et al., 2012, 2017a,b). Earlier, we showed that ICA can be effectively used to find the sources of task-related sensorimotor mu-rhythm desynchronization (Frolov et al., 2012) as well as to extract the patterns of activity of three other brain areas involved in motor imagery (Frolov et al., 2017a). However, these results were obtained using a single ICA method. In this paper, we investigate the results of the application of several source separation methods for processing our records. The methods are compared in terms of how many shared components they provide as well as the task specificity of EEG patterns they find. We are mainly focused on studying sources that have clear physiological interpretations.

In Delorme et al. (2012), it was shown that physiologically meaningful components are dipolar. Results were obtained by using several ICA methods to decompose EEG recordings corresponding to visual memory tasks performed. A component being dipolar means that its topographic weight map, which can be looked upon as a scalp potential distribution, is adequately approximated by the distribution resulting from a single current dipole. Note that EEG being a result of the superposition of multiple dipolar source potentials at a time is an accepted model of the EEG origin (Niedermeyer and Da Silva, 2005). The fact that a component is dipolar implies that it results from well-localized changes of electric activity in the brain, allowing it to be attributed to a certain brain area activation or deactivation.

Although the areas the activity of which results in the appearance of dipolar components are typically rather small (about several millimeters in diameter), they significantly impact the EEG signal recorded by all of the electrodes. That is why the main task of the decomposition method is to unmix the signal components. The unmixing done by the ICA and other BSS methods is often based on reducing the mutual information between the components (Hyvärinen et al., 2004). That is why mutual information reduction (MIR) is another criterion of method comparison.

Another indication of a component's relevance is the repeatability of its occurrence among all the subjects and records. The components reflecting common or task-specific EEG phenomena are expected to be found in most of the recordings. That is why a cluster analysis was applied to the topographic maps and power spectral densities of the components found. The results allow the evaluation of the rate at which a certain phenomenon can be discovered by the methods tested.

The paper is organized as follows. The next section provides information on the dataset and participants, as well as details of the 16 blind and non-blind signal decomposition methods used in this work. The measures of both similarity between the methods and similarity of the extracted components are described. The algorithms for estimating the task-specificity of the components and for cluster analysis are given. The results of the method comparison are then represented, and clusters of the most frequently encountered components are shown together with the cluster statistics. The results obtained are discussed, and possible physiological interpretation of the extracted components is given.

## Methods

We used the data from the BCI experiments carried out at the Institute for the Higher Nervous Activity and Neurophysiology of the Russian Academy of Science. The study was approved by the Institute's ethical committee.

### Participants

Twenty-three volunteers (7 females and 16 males) aged from 21 to 36 (26 ± 4) participated in the experiments. The volunteers were right-handed according to the Edinburgh Handedness Inventory and had no reported neurological or other disorders. All of them gave written informed consent.

None of the participants had an experience of controlling a BCI. The ability of the participants to perform motor imagery was not assessed prior to the experiments.

### Experimental Procedure

The original EEG dataset for the work was obtained using the same procedure as described in earlier works (Frolov et al., 2012, 2017a). Each subject had to sit relaxed, looking at the center of the screen or had to imagine flexion of either his left or right hand according to visual cue presented on the screen. Each task had to be performed for 10 s, with a 2 s preparation period. There were 20 cue presentations for each hand motor image, which were split into blocks containing 2 cues for each hand. In each block, the cues were given in a random order. Each motor imagery task was preceded by the relaxation period.

There were from 10 to 20 experimental days (14 ± 3) for each subject, with 1–3 day gaps possible between the sessions.

The EEG data were recorded using the NVX52 acquisition device (Medical Computer Systems, Russia) with 32 electrodes placed according to the 10–10 system. The data were band-pass filtered with a 5–30 Hz Butterworth filter as well as a 50 Hz notch filter to suppress power line interference.

### EEG Classification

The EEG data were classified with a Bayesian classifier under the assumption that the signal has a multivariate Gaussian distribution with a zero mean and a different covariance matrix for each task classified:

(1) $$\begin{array}{l} \text{P}\left(\text{x}|i\right)=\frac{1}{\sqrt{2\pi \text{ det }{\text{C}}_{i}}\;}\text{exp }\left(-\frac{{\text{x}}^{\text{T}}{\text{C}}_{i}^{-1}\text{x}}{2}\right), \end{array}$$

where **C**_*i*_ is a covariance matrix of the EEG corresponding to the ith task, and **x** is an EEG sample. According to the Bayes rule, the probability that a given sample will correspond to the ith class is proportional to *P* (**x**|*i*)*P*(*i*), where *P*(*i*) is the probability of the ith task being cued. Thus, for a signal epoch logarithm of the probability of all its samples to correspond to the ith class is proportional to $\frac{1}{n_{t}}\left(-\Sigma_{t}{\text{x}}_{t}^{\text{T}}{\text{C}}_{i}^{-1}{\text{x}}_{t}\right)-\text{ln det }{\text{C}}_{i}+\text{ln }P\left(i\right)+const$, where summation is performed over all the epoch samples, *n*_*t*_ is the epoch length in samples, and the constant term is independent of both the signal and the class number. Since $\frac{1}{n_{t}}\left(-\Sigma_{t}{\text{x}}_{t}^{\text{T}}{\text{C}}_{i}^{-1}{\text{x}}_{t}\right)$ can be substituted with trace$\left(\text{cov}\left(\text{X}\right){\text{C}}_{i}^{-1}\right)$, where cov(**X**) is the epoch covariance estimate under the assumption of a zero mean, the class number is

(2) $$\begin{array}{l} i_{class}\left(\text{X}\right)=\underset{i}{\text{argmax }}\left(\text{ln }P\left(i\right)-\text{trace }\left(\text{cov}\left(\text{X}\right){\text{C}}_{i}^{-1}\right)-\text{ln det }{\text{C}}_{i}\right) \end{array}$$

We used a window of 1 s length sliding with a 100 ms shift to present the feedback during the sessions. The probabilities *P* (*i*) were estimated based on the cue durations. The classifier was updated after each block of cues, with no feedback during the first block.

### Signal Decomposition

All of the blind and non-blind source separation methods used can be, in general, represented in the form of multivariate linear decomposition with an optional noise term.

(3) $$\text{X}={\text{a}}_{1}\xi_{1}+\ldots +{\text{a}}_{n}\xi_{n}+\text{N}=\text{A}\Xi +\text{N},$$

where **X** is the decomposed signal in matrix form, **a**_*i*_, *i* = 1, …, *n*, are columns of **A** that define the source (component) weights representing the source topographic maps, ***ξ***_*i*_, *i* = 1, …, *n*, are row vectors of the source activities, *n* is the number of EEG channels, and is the optional noise term. Matrix **A** is called the mixing matrix, and its inverse, **W**, is called the unmixing matrix. The invertibility of **A** is based on the assumptions that there are as many components as there are signal channels and that the signal correlation matrix has full rank. These assumptions are in general not required but are common for linear source separation methods (Cichocki and Amari, 2002; Hyvärinen et al., 2004), and all of the decomposition methods used in the paper were derived under these assumptions.

Usually, the signal is whitened before searching for decomposition (3) so that the covariance matrix of **X** becomes an identity matrix. Also, it is well-known that the component activity variances are not defined, thus requiring the application of some constraints when the decomposition is computed. That is why we shall further consider the decomposed signal to be whitened, i.e.

(4) $$\begin{array}{l} \text{X}=\text{V}\text{Z}, \end{array}$$

where **V** is a matrix such that **V**cov(**X**)**V**^T^ = **I**. If we also suppose that all ***ξ***_*i*_, *i* = 1, …, *n*, have unit variance, then the mixing matrix for **Z** is orthogonal, **Z** = **UΞ**, **UU**^T^ = **I**, and the EEG mixing matrix is **A** equals **VU**.

We used 16 source separation methods to obtain the decomposition (3).

**PCA**, or Principle Component Analysis, is a classic example of a technique for obtaining the decomposition (3). It uses Singular Value Decomposition to diagonalize the signal covariance matrix. Usually, the components with too much or too little variance are discarded. If one rather chooses to keep all of the components under the constraints that their variances are equal to 1, this will become signal whitening. In this case, **U** = **I**, and the unmixing matrix is not orthonormal.

**KURT** is an ICA method based on maximizing the difference of the component distributions from normal. The difference is measured by the absolute value of the component excess kurtosis, which serves as a cost function for iterative component search (Delfosse and Loubaton, 1995; Hyvärinen et al., 2004), i.e., the columns of unmixing matrix **W** are estimated sequentially by maximizing the excess kurtosis for ${\text{w}}_{i}^{\text{T}}\text{Z}$ under the assumption that **W** is orthogonal.

**CUMUL** is an ICA method using the signal non-stationarity as the criterion of statistical independence (Hyvarinen, 2001). The non-stationarity is measured by the fourth-order cumulant:

(5) $$\begin{array}{l} {\text{cum}}_{4}\;(x)=\;E\left\{x^{2}\;(t)\;x^{2}\;(t-\tau )\right\}-E\{x^{2}\;(t)\}\;E\left\{x^{2}\;(t-\tau )\right\}\\ \begin{array}{l} \;-2{E\left\{x\left(t\right)x\left(t-\tau \right)\right\}}^{2}. \end{array} \end{array}$$

The decomposition seeks to maximize the sum of the source cumulants. The proof of non-stationarity maximization being a criterion of component independence can be found in Hyvärinen et al. (2004), chapter 18. The time lag τ serves as the method parameter. In our experiment, it was set to 100 ms to extract the components with dominant alpha-band frequencies.

**FastICA** is an ICA method in which mutual information reduction between the components serves as the statistical independence criterion. The mutual information between the components ***ξ***_*i*_, *i* = 1, …, *n*, is defined as

(6) $$\begin{array}{l} I\left(\;\xi_{1},\ldots ,\xi_{n}\right)=\underset{i}{\sum }\text{H}\left(\xi_{i}\right)-H\left(\Xi \right), \end{array}$$

where *H* (·) denotes entropy. The entropy of linear transformation is *H* (**WZ**) = *H* (**Z**) + |det(**W**)|. Under the assumption of **Z** being whitened and the components having unit variance, **W** is orthogonal, which implies that det (**W**) = 1 and *H* (**WZ**) does not depend on det (**W**). As a result, $H\left({\text{w}}_{i}^{\text{T}}\text{Z}\right)$ can be used as a cost function for sequential search of the unmixing matrix columns **w**_*i*_.

Since computing the entropy requires knowledge on the signal distribution, different parametric approximations are used. FastICA uses approximation in the form

(7) $$\begin{array}{l} H\left(x\right)\propto const-{\left[E\;\left\{g\;\left(x\right)\right\}-E\;\left\{g\;\left(v\right)\right\}\right]}^{2}, \end{array}$$

where *g* (*x*) is a non-linear function and *v* is a random normally distributed variable with unit variance. FastICA can be computed using different non-linearities (Bingham and Hyvärinen, 2000; Hyvärinen et al., 2004). In this work, we used two functions, namely, tanh(*x*) and *x*exp(−*x*^2^/2). The corresponding methods are denoted as FastICAT and FastICAG.

**RunICA**, or extended infomax, is an ICA method that is similar to FastICA. The method takes into account both super-and sub-Gaussian distributions by applying two different non-linearities, *g*_+_ (*x*) = −2 log cosh (*x*) and $g_{-}\left(x\right)=\text{log cosh }\left(x\right)-x^{2}/2$, and switching between them. Originally, the infomax algorithm was introduced by Bell and Sejnowski (1995) based on a neural network approach to maximize the entropy of the neural network output. It was later reformulated in terms of parametric non-linearities for source entropy estimation (Lee et al., 1999).

**AMICA**, which stands for Adaptive Mixture Independent Component Analysis, is an ICA method based on the assumption that all EEG components have generalized Gaussian mixture distributions

(8) $$\begin{array}{l} p\left(\xi_{i}\right)=\underset{j=1,\ldots ,k}{\sum }\alpha_{ij}q\left(\xi_{i},\rho_{ij},\mu_{ij},\beta_{ij}\right), \end{array}$$

(9) $$\begin{array}{l} q\left(\xi ,\rho ,\mu ,\beta \right)=\frac{\rho }{2\beta \Gamma \left(1/\rho \right)}\text{exp}\left(-|\frac{\xi -\mu }{\beta }{|}^{\rho }\right). \end{array}$$

AMICA can also segment the signal into several parts corresponding to different models (8) when the number of the models is set to higher than 1. All distribution parameters, unmixing matrix coefficients, and model scores (in the case of several models) are estimated using the expectation-maximization algorithm (Palmer et al., 2006, 2008, 2012). In our work, we compared AMICA with the default settings (2 models, 2 distributions in mixture) to AMICA with two models and Gaussian distributions with means fixed to zero (2 models, 2 distributions in mixture, $\rho_{i,j}^{l}=2$, $\mu_{i,j}^{l}=0$, where *l* indicates the model, *l* = 1, 2, and *i* = 1, …, *n, j* = 1, 2), and to AMICA with a single model and Gaussian distributions with means fixed to zero (1 model, 2 distributions in mixture, ρ_*ij*_ = 2, μ_*ij*_ = 0, *i* = 1, …, *n, j* = 1, 2). The two latter cases will be denoted as AMICA1 and AMICA2, respectively.

**PWCICA** is an ICA method mapping the signal into a complex domain using the first derivative (rate) of the signal as its imaginary part. The algorithm is described in Ball et al. (2016). It uses the complex variant of FastICA in order to find decomposition (3) in the complex domain (Bingham and Hyvärinen, 2000). After that, the real-valued unmixing matrix is computed using the algorithm provided in Ball et al. (2016).

**SOBI**, or Second Order Blind Identification, is a blind source separation method that uses joint diagonalization to remove signal cross-covariance for a set of defined time lags (Belouchrani et al., 1997). Particularly, a set of time lags δ_*k*_ is specified, and corresponding cross-covariance matrices are estimated:

(10) $$\begin{array}{l} {\text{C}}_{k}=\text{E}\;\left(\text{X}\;\left(t\right),\text{X}\left(t-\delta_{k}\right)\right), \end{array}$$

Next, the unmixing matrix **W** is found by joint diagonalization of the estimated cross-covariance matrices:

(11) $$\begin{array}{l} \text{W}=\underset{\text{M}}{\text{argmin}}\left(\Sigma_{k}\|\text{M}{\text{C}}_{\text{k}}{\text{M}}^{\text{T}}{\|}_{\text{off}}\right) \end{array}$$

where ||·||_*off*_ denotes the Euclidean norm of the off-diagonal part of a matrix. The norm of the unmixing matrix column is constrained to be equal to 1. There exist several algorithms for solving the problem (11). We used one proposed in Ziehe et al. (2004) to obtain the solution.

**CSP**, or Common Spatial Patterns (Ramoser et al., 2000; Bashashati et al., 2007, 2015), is a non-blind decomposition method, in contrast to the source separation techniques described above. This method utilizes the information on signal segmentation with respect to the experimental tasks in order to find the decomposition (3). The method is used for feature extraction in two-class separation problems. The decomposition is sought that maximizes the component variance ratio for the states classified under the assumption that total component variance is fixed. This is equivalent to finding matrix **W** such that

(12) $$\begin{array}{l} \left\{\begin{array}{c} \text{W}{\text{C}}_{1}{\text{W}}^{T}={\text{D}}_{1}\\ \text{W}\left({\text{C}}_{1}+{\text{C}}_{2}\right){\text{W}}^{T}=\text{I} \end{array}\right\}\;, \end{array}$$

where **C**_*i*_ is the i-th class covariance matrix and **D**_1_ is a diagonal matrix. The equations (12) have an explicit solution where **W** and **D**_1_ result from singular-value decomposition (SVD) of the matrix ${\text{D}}^{-1/2}\;{\text{U}}^{\text{T}}{\text{C}}_{1}\text{U}{\text{D}}^{-1/2}$ with orthogonal matrix **U** and diagonal matrix **D** given by SVD of **C**_1_ + **C**_**2**_. We used several CSP decompositions in this work, namely, CSP12 comparing relaxation and left hand motor imagery, CSP13 comparing relaxation and right hand motor imagery, CSP23 comparing left with right hand motor imagery, and CSP1X comparing relaxation and both left and right hand motor imagery. Although equations (12) are solved using signal epochs corresponding to the selected tasks only, the unmixing matrix was used to estimate the activity of the components at all other time moments.

**MCSP** is a generalization of the CSP method for multiple-class problems. There are several methods of generalization (Lee et al., 2005; Bashashati et al., 2015). The most frequent approaches are to either compute all pairwise CSP and combine the corresponding components into a new signal to be classified or to use classifier voting or a tree of some sort when only two classes are compared at each step (e.g., relaxation vs. motor imagery with subsequent discrimination between the hands if the motor imagery was recognized). However, these techniques would not give us any additional information in terms of sources, as we have already considered all pairwise CSP decompositions for our experimental tasks, as well as the CSP1X comparing relaxation and motor imagery. That is why we have decided to use a different approach. Problem (12) was generalized so as to find **W** such that

$$\{\begin{array}{c} \text{W}{\text{C}}_{1}{\text{W}}^{\text{T}}={\text{D}}_{1}\\ \text{W}{\text{C}}_{2}{\text{W}}^{\text{T}}={\text{D}}_{2}\\ \text{W}({\text{C}}_{1}+{\text{C}}_{2}+{\text{C}}_{3}){\text{W}}^{\text{T}}=\text{I} \end{array}$$

The equations are written for the case of three tasks, but the generalization for the case of an arbitrary number of classes is straightforward. The problem in general has no explicit solution and can be solved numerically using any joint diagonalization technique. The joint diagonalization algorithm was the same as was used to perform the SOBI decomposition (Ziehe et al., 2004). It should be noted that unlike the two-class CSP, component variance ratio maximization (or minimization) for different classes is not guaranteed, but if any component has high (small) variance for a certain class, it will have small (high) variance for other classes.

### Mutual Information Reduction

The mutual information reduction (MIR) provided by the decomposition methods is defined as

(13) $$\begin{array}{l} \text{MIR}=\text{I}\;(x_{1},\ldots x_{n})-\text{I}\left(\xi_{1},\ldots ,\xi_{n}\right), \end{array}$$

where the mutual signal information *I* is given by equation (6). Using (6), we get

(14) $$\begin{array}{l} \text{MIR}=\Sigma_{i}\text{H}\left(x_{i}\right)-\text{H}\left(\text{X}\right)-\Sigma_{i}\text{H}\left(\xi_{i}\right)+H\left(\Xi \right) \end{array}$$

Since **X** = **AΞ**,

(15) $$\begin{array}{l} \text{H}\left(\text{X}\right)=\text{H}\left(\Xi \right)+\text{log}|\text{det }\text{A}| \end{array}$$

and thus

(16) $$\begin{array}{l} \text{MIR}=\Sigma_{i}\text{H}\left(x_{i}\right)-\Sigma_{i}\text{H}\left(\xi_{i}\right)+\text{log}|\text{det }\text{A}| \end{array}$$

The entropy of each individual channel and component was estimated and bias-corrected as in Delorme et al. (2012).

### Dipolar Component Selection

The decomposition (3) shows that the contribution of each component to the signal registered by the EEG electrodes is given by the weight vector, which can be treated as potential distribution over the scalp surface. This allows the source of electrical activity represented by the component to be localized. As has been shown for the case of visual memory tasks, the physiologically meaningful components tend to have dipolar distributions of their weights, i.e., the distributions that could be adequately approximated by a single current dipole inside a head model (Delorme et al., 2012). We had neither anatomical magnetic resonance imagery (MRI) scans nor digitized electrode positions for most of our subjects. This is why a standardized head model based on the ICBM MNI atlas (Fonov et al., 2009, 2011) was used for estimating the dipolarity of the component weights. The dipolarity was computed as a residual variance of the best single dipole fit for the corresponding distribution. The fit was considered acceptable if the residual variance did not exceed 10%.

### Shared Components

The number of the same components found by different decomposition methods can be used as a measure of similarity between the methods. On the other hand, the more methods reveal the component, the less likely it is that the component is an artifact of a certain mathematical procedure underlying the decomposition algorithm. That is why we focused our attention on the components found by different methods.

Two component similarity measures were used to determine which components were shared between the methods. The first measure was the absolute value of the cosine between normalized component weight vectors given by the corresponding mixing matrix columns. The second one was the absolute value of the Pearson correlation between the component activities. Two thresholds were chosen for the measures. The components were considered the same when both measures exceeded the corresponding thresholds. We have chosen 0.9 as the threshold for the topographic map similarity and 0.8 as the threshold for activity correlation.

When the number of shared components is obtained, the similarity between the decomposition methods can be measured as

(17) $$\begin{array}{l} \text{si}{\text{m}}_{\text{ij}}=\frac{n_{s}}{n_{i}+n_{j}-n_{s}}, \end{array}$$

where *n*_*s*_ is the number of shared components, and *n*_*i*_ and *n*_*j*_ are the numbers of components obtained by the i-th and j-th methods, respectively. The similarity measure varies from 0, when there are no shared components, to 1, when the methods have provided identical decomposition. Also, only the dipolar components can be considered when the similarity (17) is calculated, allowing non-dipolar components that are unlikely to have physiological meaning to be discarded. The number of methods that found a component minus one will be called the component rank. The component rank equals 0 when it is found by only one method and 15 when it is found by all of the 16 methods considered.

### Classification Accuracy and the Component Specificity

Given a set of the EEG components, their activity can be used for estimating the accuracy of discriminating between the experimental tasks. This estimate can serve as a measure of components' specificity for the performed tasks, allowing the task-specific EEG patterns to be extracted and both artifacts and irrelevant EEG activity to be removed.

We used a greedy algorithm to find the task-specific components as follows. Only the dipolar components were considered. At the first step, classification accuracy was estimated for each set containing one or two components. The best set was then selected. The remaining components were added to the set one by one so that the new set would provide the highest classification accuracy. The components from the set for which the classification accuracy was maximal (over all of the steps) were considered as the task-specific components. The classification was tested by 120 trials of cross-validation where 7 blocks were chosen for the training set and the 3 remaining blocks were used as the testing set.

The accuracy of task classification was measured by Cohen's kappa index, κ (Vieira et al., 2010), which was calculated from the elements of the confusion matrix (*g*_*ij*_) resulting from the cross-validation. The element *g*_*ij*_ counts how many times the i-th task was recognized by the classifier under the condition that the classified EEG epoch corresponded to the j-th cue. Given *g*_*ij*_, the κ index is

(18) $$\begin{array}{l} \kappa =\frac{g_{0}\sum_{i}g_{ii}-\sum_{j}g_{j}^{2}}{g_{0}^{2}-\sum_{j}g_{j}^{2}}, \end{array}$$

where *g*_0_ is the sum of all of the elements of the confusion matrix, $\underset{i}{\sum }g_{ii}$ is the sum of all diagonal elements of the confusion matrix, and *g*_*j*_ is the sum of all of the elements of the j-th column of the confusion matrix. The index varies from −1 to 1 (perfect classification) and equals 0 in case of random classification.

### Component Clustering

The decomposition (3) gives at least as many components as there are electrodes for each of the methods used. Consequently, the number of components computed for all experimental recordings is too high to check the components manually. In order to investigate EEG patterns most typical for our experimental tasks, we used cluster analysis to group the components according to their similarity. The clustering was performed using the Attractor Neural Network with Increasing Activity (ANNIA), which was originally proposed for Boolean factor analysis (Frolov et al., 2007). Adaptation of the technique for cluster analysis is described in Bobrov et al. (2014). When the ANNIA is used for clustering, the strength of synaptic connections between the neurons of the network is determined by the similarity between the corresponding elements rather than by Hebbian learning. The stopping criterion is based on a threshold that specifies the minimal average similarity between the elements of a cluster. In Bobrov et al. (2014), the ANNIA was used to group the components with respect to their topographic map similarity. In this paper, we have also accounted for the component activity similarity using the correlation coefficient between the components' activity power spectral densities estimated for each of the experimental tasks. Spectral analysis was used since the components obtained from different recordings were compared, in contrast with the shared components search described earlier. Both the component similarity measures considered vary from 0 to 1, and their mean was taken as the similarity measure for ANNIA.

## Results

### Comparison of the Decomposition Methods

The methods were compared according to the criteria described in the previous section. The percentage of dipolar components found by each method is presented in Table 1 (median values and quartiles are presented). The table also contains average values of mutual information reduction values for each of the methods. The *p*-values resulting from pairwise comparison of the percentage of the dipolar components found are presented in the upper diagonal part of Table 2. The values were obtained using a Wilcoxon test of the values pooled from all of the participants and sessions with subsequent Benjamini-Hochberg correction (initial significance level: 0.05; resulting critical value: 0.0429). The results indicate that the PWCICA and AMICA methods provide more dipolar components than the other methods. The difference is significant for the AMICA methods and insignificant when PWCICA is compared to other BSS methods. The PCA and CSP methods extracted significantly fewer dipolar components than the other methods.

**Table 1:** Percentage of dipolar components found and the method MIR.

**Method**	**% of dipolar components**	**MIR, bits/(sec·chan)**
PCA	4.35 [2.17; 7.50]	63.56 [51.89; 77.34]
KURT	15.39 [6.98; 27.50]	70.15 [58.97; 83.69]
CUMUL	12.77 [6.38; 22.61]	70.96 [59.21; 84.81]
FastICAT	15.63 [8.51; 27.50]	68.24 [57.28; 82.96]
FastICAG	15.22 [8.33; 27.33]	67.58 [56.60; 83.75]
RunICA	10.87 [3.54; 25.00]	74.92 [65.21; 86.47]
AMICA	25.53 [11.11; 38.96]	68.98 [57.47; 83.99]
AMICA1	21.74 [12.50; 32.55]	69.23 [58.11; 83.95]
AMICA2	27.50 [15.38; 40.00]	66.74 [55.88; 80.30]
PWCICA	19.57 [6.38; 31.25]	67.42 [56.00; 81.38]
SOBI	15.22 [6.67; 26.67]	71.52 [58.80; 84.88]
CSP12	4.26 [1.37; 13.68]	62.99 [50.11; 78.13]
CSP13	4.17 [2.08; 12.74]	63.24 [48.94; 77.74]
CSP1X	4.26 [1.91; 14.97]	63.72 [50.34; 77.87]
CSP23	2.22 [1.23; 10.00]	63.18 [48.82; 78.13]
MCSP	6.90 [2.17; 17.95]	63.89 [51.40; 77.78]

**Table 2:** The *p*-values of pairwise Wilcoxon testing of the percentage of the dipolar components found (upper triangle part) and the pattern classification accuracy (lower triangle part).

	**MCSP**	**CSP23**	**CSP12**	**CSP1X**	**CSP13**	**AMICA1**	**AMICA**	**AMICA2**	**PWCICA**	**SOBI**	**FastICAT**	**KURT**	**FastICAG**	**CUMUL**	**RunICA**	**PCA**
**MCSP**		<1e-4	<1e-4	<1e-4	<1e-4	<1e-4	<1e-4	<1e-4	<1e-4	<1e-4	<1e-4	<1e-4	<1e-4	<1e-4	0.0053	<1e-4
**CSP23**	0.0463		0.0312	0.0053	0.0045	<1e-4	<1e-4	<1e-4	<1e-4	<1e-4	<1e-4	<1e-4	<1e-4	<1e-4	<1e-4	0.0037
**CSP12**	**0.1150**	**0.6045**		**0.5743**	**0.5525**	<1e-4	<1e-4	<1e-4	<1e-4	<1e-4	<1e-4	<1e-4	<1e-4	<1e-4	<1e-4	**0.8583**
**CSP1X**	**0.0352**	**0.9696**	**0.5822**		**0.9963**	<1e-4	<1e-4	<1e-4	<1e-4	<1e-4	<1e-4	<1e-4	<1e-4	<1e-4	<1e-4	**0.3943**
**CSP13**	0.0094	**0.6366**	**0.3041**	**0.6029**		<1e-4	<1e-4	<1e-4	<1e-4	<1e-4	<1e-4	<1e-4	<1e-4	<1e-4	<1e-4	**0.5076**
**AMICA1**	<1e-4	0.0231	0.0056	0.0175	**0.0628**		0.0323	<1e-4	0.0004	<1e-4	<1e-4	<1e-4	<1e-4	<1e-4	<1e-4	<1e-4
**AMICA**	<1e-4	0.0056	0.0015	0.0046	0.0181	**0.6061**		**0.0673**	<1e-4	<1e-4	<1e-4	<1e-4	<1e-4	<1e-4	<1e-4	<1e-4
**AMICA2**	**0.0682**	**0.7721**	**0.5554**	**0.8452**	**0.8395**	**0.0940**	**0.0457**		<1e-4	<1e-4	<1e-4	<1e-4	<1e-4	<1e-4	<1e-4	<1e-4
**PWCICA**	<1e-4	0.0007	<1e-4	0.0005	0.0027	**0.2870**	**0.6421**	0.0076		**0.0937**	**0.5838**	**0.1890**	**0.2345**	0.0008	<1e-4	<1e-4
**SOBI**	<1e-4	0.0006	<1e-4	0.0003	0.0016	**0.2197**	**0.5266**	0.0055	**0.8586**		**0.2654**	**0.7172**	**0.6611**	0.0350	0.0003	<1e-4
**FastICAT**	<1e-4	<1e-4	<1e-4	<1e-4	0.0002	**0.0825**	**0.2646**	0.0012	**0.4937**	**0.6218**		**0.4582**	**0.5342**	0.0013	<1e-4	<1e-4
**KURT**	<1e-4	<1e-4	<1e-4	<1e-4	<1e-4	**0.0758**	**0.2594**	0.0009	**0.4703**	**0.5728**	**0.9670**		**0.9049**	0.0158	<1e-4	<1e-4
**FastICAG**	<1e-4	<1e-4	<1e-4	<1e-4	<1e-4	**0.0515**	**0.2027**	0.0007	**0.3798**	**0.5108**	**0.8375**	**0.9007**		0.0094	<1e-4	<1e-4
**CUMUL**	<1e-4	<1e-4	<1e-4	<1e-4	<1e-4	0.0145	**0.0839**	<1e-4	**0.1834**	**0.2308**	**0.5076**	**0.5392**	**0.6238**		**0.0526**	<1e-4
**RunICA**	<1e-4	<1e-4	<1e-4	<1e-4	<1e-4	0.0024	0.0202	<1e-4	**0.0391**	**0.0659**	**0.1537**	**0.1792**	**0.2098**	**0.4488**		<1e-4
PCA	<1e-4	<1e-4	<1e-4	<1e-4	<1e-4	<1e-4	<1e-4	<1e-4	<1e-4	<1e-4	<1e-4	<1e-4	<1e-4	0.0002	0.0100	
On-line	<1e-4	<1e-4	<1e-4	<1e-4	<1e-4	<1e-4	<1e-4	<1e-4	<1e-4	<1e-4	<1e-4	<1e-4	<1e-4	<1e-4	<1e-4	<1e-4

*The values indicating insignificant difference are in bold*.

Pairwise Wilcoxon comparison of the MIR values with Benjamini-Hochberg correction (initial significance level: 0.05; resulting critical value: 0.0275) shows that the ICA methods provide significantly higher mutual information reduction than the PCA and CSP methods. The difference between ICA and other methods in terms of MIR was insignificant, as was the difference between the PCA and CSP methods.

**Figure 1:**
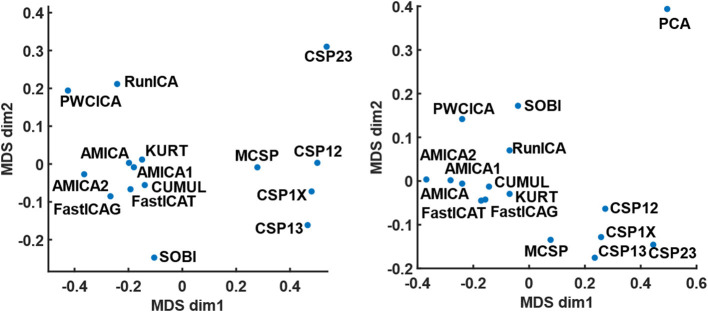
The results of multidimensional scaling according to method similarity. The mapping on the left panel is obtained for the case where all the components were considered. The mapping on the right panel is obtained for the case where only the dipolar components were considered. The PCA point is omitted from the left panel due to its remoteness from all other points.

The method similarity was estimated by the fraction of shared components according to (17), providing a method similarity matrix. The similarity matrices for the cases when either all or only dipolar components were considered were used for mapping the method onto a 2D plane with a multidimensional scaling technique. The results of the mapping are shown in Figure 1. Apparently, the methods tend to group according to the decomposition criteria, one group containing the ICA methods except PWCICA, and another group containing the CSP methods. SOBI and PWCICA are distant from both groups, while the PCA is as an outlier.

**Figure 2:**
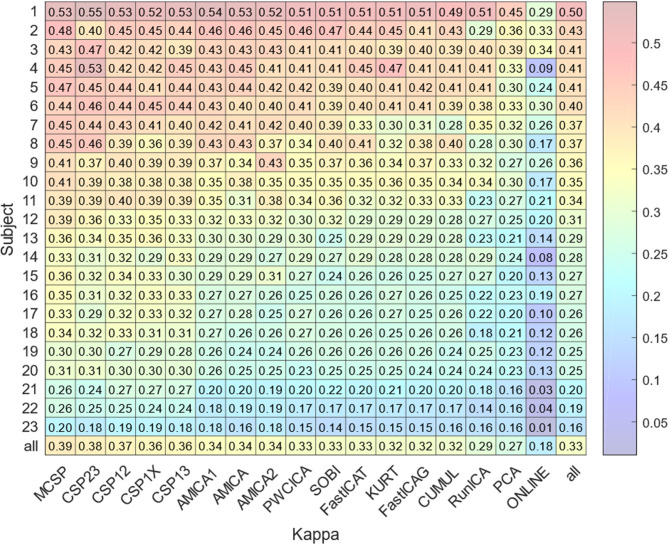
Average accuracy of the experimental task classification for all subjects and methods. The bottom row marked “all” contains the values averaged over all the methods except on-line. The rightmost column marked “all” contains the values averaged over all the subjects. The bottom right value is obtained by averaging over all subjects and methods.

The results for classification accuracy, obtained after searching for the task-specific components, are shown in Figure 2. Mean κ values are shown for each subject and each method. The methods are sorted according to the κ value averaged over all the subjects. The subjects are sorted according to the κ value averaged over all the methods. The lower diagonal part of Table 2 contains *p*-values resulting from the pairwise comparison of the κ values for different methods. The values were obtained using Wilcoxon test of the values pooled from all of the participants and sessions with Benjamini-Hochberg correction (initial significance level: 0.05; resulting critical value: 0.320). The CSP methods provide patterns that are significantly better classified than those found by the other methods. The PCA patterns are significantly worse-classified than the patterns found by the other methods. The task-specific component search yielded accuracy values significantly exceeding those obtained during on-line BCI operation, which is likely due to discarding irrelevant, artifact, and noisy components.

### Component Clustering

**Figure 3:**
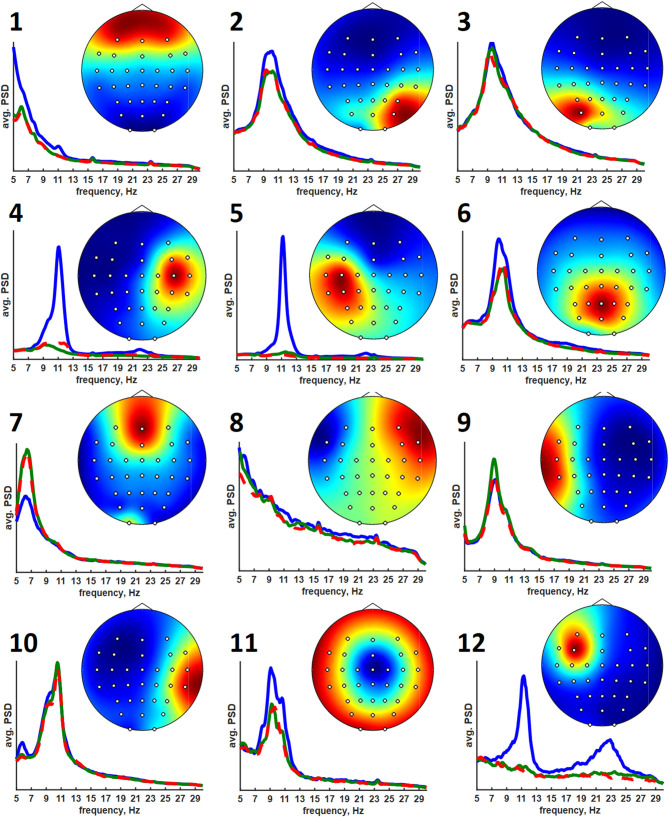
Average topographic maps and power spectral densities of the first 12 component clusters. The PSDs are plotted in the 5–30 Hz range. Blue lines correspond to relaxation, green lines correspond to left hand motor imagery, and red lines correspond to right hand motor imagery.

The clusters obtained using the ANNIA were sorted according to their component occurrence, i.e., the percentage of the experimental session in which the components of the cluster were found. Figure 3 shows topographic maps and activity power spectral densities (PSD) for the components of the first 12 clusters. The maps and PSDs were obtained by averaging over all of the cluster components regardless of the subject or session. Table 3 presents the occurrence, average dipolarity, average rank, and specificity of the components of each cluster. As stated above, the component dipolarity was measured by the residual variance of the topographic map fit with a potential distribution resulting from a single current dipole, and the component rank is the number of methods that found the component minus one. The specificity was calculated as the percentage of cases when the component was included in the best set of components (the set search is described in the Methods section) among the cases when the component was found.

**Table 3:** The cluster statistics.

**Cluster**	**1**	**2**	**3**	**4**	**5**	**6**	**7**	**8**	**9**	**10**	**11**	**12**
Occurrence, %	71	70	61	52	52	48	39	38	35	30	26	22
Dipolarity, %	2	9	8	8	4	3	2	3	9	8	3	4
Rank	3.74	2.24	1.78	1.62	3.53	2.83	2.66	1.48	1.06	1.78	1.92	2.23
Specificity, %	21	10	8	24	59	61	34	10	16	18	26	41

It should be mentioned that AMICA restricted to only Gaussian distributions in mixtures performed as well as AMICA with the generalized super-Gaussian mixtures, suggesting that dipolar components obtained from band-pass filtered EEG signal have activity the distribution of which can be adequately approximated with a mixture of two Gaussians.

## Discussion

In the EEG\MEG field, and, particularly, in BCI research, ICA methods are mostly used for artifact removal during signal preprocessing or epoch classification. They are applied for eliminating ocular (Höller et al., 2013; Dharmaprani et al., 2016; Sarin et al., 2020), motion (Zhou et al., 2016; Kobler et al., 2019), and muscle (Höller et al., 2013; Dharmaprani et al., 2016) artifacts. The most popular method used for artifact suppression is RunICA (extended infomax), likely due to its incorporation into EEGLAB (Delorme and Makeig, 2004) and BrainStorm (Tadel et al., 2011) software. The ICA methods are also believed to provide effective spatial filters for BCI feature selection (Kachenoura et al., 2007; Lotte et al., 2018; Xiaopei et al., 2019). The main limitation to using the methods is that they require a substantial amount of data to converge to meaningful decomposition, and many of the algorithms have high computational costs to implement on-line. These issues, as well as the nature of the algorithm used to automatically select the most specific components, limit the on-line implementation of the procedure proposed in this paper. The procedure is rather intended for assessing the overall task-specificity of the EEG signal and adequately arranging experimental recordings and subjects with respect to the BCI control efficiency. Performing the cluster analysis allows the most frequent and typical EEG patterns in the processed recordings to be extracted with no need to choose a single decomposition method. The best method may differ when different criteria are applied, such as the number of the dipolar components found, the mutual information reduction, or the accuracy of classification of the extracted patterns.

The method comparison shows that ICA methods provide results similar to each other despite the difference in the independence criteria underlying the algorithms. These methods find significantly more dipolar components and provide significantly higher MIR than CSP or PCA, with the best results being for AMICA and PWCICA. PCA also has the lowest number of components shared with the other methods, as indicated by the results of multidimensional scaling. The maximal similarity between the PCA and other methods, estimated according to (17), was five times less than the minimal similarity between the other methods when all of the components were considered and two times less when only the dipolar components were considered. On the other hand, our results indicate that non-blind CSP methods find more of the separable activity patterns, much like in Xiaopei et al. (2019), where three ICA methods were compared to CSP in terms of providing the best spatial filters for a BCI classifier. However, unlike our case, no significant difference between RunICA and CSP was observed in Xiaopei et al. (2019). CSP outperformed the ICA methods, likely due to accounting for the signal segmentation with respect to the experimental tasks. Thus, CSP decomposition is likely to find the dipolar components with task-specific activity, while it is poor at extracting other task-irrelevant dipolar sources. However, finding irrelevant but physiologically plausible sources may be useful for proving that BCI works based on the activity of the targeted brain areas. E.g., it may be important to establish that motor imagery BCI used for post-stroke rehabilitation worked because of the activity of the motor areas and not just due to eye movements or differences in concentration level.

The results of cluster analysis agree with those obtained earlier for fewer healthy subjects and patients with subcortical lesions (Frolov et al., 2017a). In previous work, the components relevant to controlling a hand exoskeleton via BCI were found to correspond to the sources of sensorimotor mu-rhythm located at the bottom of the central sulci of the left (SIL) and right (SIR) hemispheres, alpha-rhythmic activity in the precuneus (Prc), and alpha- and beta- activity in both the supplementary motor area (SMA) and in the premotor cortex of the left hemisphere (PrmL). These sources correspond to clusters 5 (SIL), 6 (SIR), 3 (Prc), 11 (SMA), and 8 (PrmL). We have also found clusters corresponding to blinking (1) and eye movements (7), which sometimes exhibited task-relevant activity, e.g., the subjects often blinked more frequently during relaxation. Clusters 2 and 3 correspond to the occipital alpha rhythm, which has low task-specificity. Interestingly, the source symmetrical to PrmR was more frequent compared to the earlier results (Frolov et al., 2017a). Source 12 exhibited activity similar to the mu-rhythm, but unfortunately, the subjects for whom it was observed had no individual anatomical MRI scans, so the source could not be reliably localized. However, the SIL source was found for more than half of the recordings for which source 12 was extracted. Also, sources SIL and 12 were found together by the same method in 35% cases. These findings suggest that source 12 might have localization different from that of the SIL sources.

The average PSDs of the clustered component activities reflect the specificity presented in Table 2. The most specific components of the 5-th, 6-th, and 12-th clusters exhibit prominent rhythm desynchronization during the motor imagery. Notably, there is no evident lateralization in the average level of desynchronization between the hemispheres. There are different results on the mu-rhythm behavior in the ipsilateral hemisphere during the hand motor imagery. Our results agree with those obtained in Vasilyev et al. (2016), where ipsilateral rhythm suppression was observed. However, ipsilateral rhythm synchronization was observed in Nam et al. (2011). We suppose that the reason for the result disagreement lies in the BCI protocol. It seems that active motor imagery, when maintained for a long time (from 10 s in these experiments to 20 s in our earlier NIRS study (Bobrov et al., 2016), involves activation of both hemispheres, either due to high concentration and the complexity of the task or due to the person's intention to “check” whether the requested hand movement has been imagined. The first explanation is supported by the results of Nam et al. (2011), where the ipsilateral rhythm synchronization was observed mainly in the end phase of brief motor imagery (1 s) trials, suggesting that brief and prolonged motor imagery may require different strategies. The latter explanation comes from some of the participants reporting that they had sometimes switched attention to the other hand to “make sure it isn't moving”. Note, that both phenomena (different strategy and attention switch) may be present during prolonged motor imagery and underlie the absence of the asymmetry of rhythm suppression.

Unlike the SIL, SIR, and 12th sources, other sources do not exhibit such prominent rhythm desynchronization or synchronization during the motor imagery. However, the Prm, SMA, and Prc sources are often marked as being task-specific. These areas are often reported to be relevant to motor planning, execution, and imagery. The Prm sources are likely to reflect mirror neuron system activation when the image of the motion to be executed or imagined is generated to be compared to the incoming sensory information (Rizzolatti et al., 2014). The SMA source is likely to correspond to the supplementary motor area activation. This area is considered to be involved in the motion timing, motion sequence generation, or motion suppression (Rizzolatti et al., 2014). It was reported to be more active during the motor imagery than during real motion execution, suggesting that its main role is to suppress the actual movement when motor imagery is performed (Guillot et al., 2014). The occurrence of the Prm and SMA sources is not as high as that of the Prc and SI sources. Nevertheless, we believe that these areas are activated in all of the participants but that the activations are not often prominent in the electroencephalogram and thus are harder to find, even when using advanced techniques. This supposition is supported by the results of numerous fMRI studies (Hétu et al., 2013) that report up to 34 areas being active during motor imagery.

In conclusion, we suggest the use of multi-method decomposition with subsequent component specificity estimation and clustering, focusing on the shared components. The primary methods to be used are PWCICA, AMICA, and MCSP, but utilizing other algorithms to support the findings seems to be reasonable.

## Data Availability Statement

The raw data supporting the conclusions of this article will be made available upon request by the authors, without undue reservation.

## Ethics Statement

The studies involving human participants were reviewed and approved by Ethics committee of Institute of Higher Nervous Activity and Neurophysiology of Russian Academy of Sciences. The patients/participants provided their written informed consent to participate in this study.

## Author Contributions

MI, YK, DB, and AL performed the experiments and collected the data. DB and AL designed the experimental software. PB, MI, and YK analyzed EEG data. AF and PB contributed to the interpretation of the data. AF, PB, and EB contributed to writing the manuscript. All authors approved the final version of the manuscript for submission.

## Conflict of Interest

The authors declare that the research was conducted in the absence of any commercial or financial relationships that could be construed as a potential conflict of interest.

## References

[B1] Ang K. K. Chua K. S. G. Phua K. S. Wang C. Chin Z. Y. Kuah C. W. K.. (2015). A randomized controlled trial of EEG-based motor imagery brain-computer interface robotic rehabilitation for stroke. Clin. EEG Neurosci. 46, 310–320. 10.1177/1550059414522229 24756025

[B2] Ang K. K. Guan C. Chua K. S. G. Ang B. T. Kuah C. W. K. Wang C.. (2011). A large clinical study on the ability of stroke patients to use an EEG-based motor imagery brain-computer interface. Clin. EEG Neurosci. 42, 253–258. 10.1177/155005941104200411 22208123

[B3] Bai Z. Fong K. N. Zhang J. J. Chan J. Ting K. (2020). Immediate and long-term effects of BCI-based rehabilitation of the upper extremity after stroke: a systematic review and meta-analysis. J. Neuroeng. Rehabil. 17, 1–20. 10.1186/s12984-020-00686-2 32334608 PMC7183617

[B4] Ball K. Bigdely-Shamlo N. Mullen T. Robbins K. (2016). PWC-ICA: a method for stationary ordered blind source separation with application to EEG. Comput. Intell. Neurosci. 2016:20. 10.1155/2016/9754813 27340397 PMC4909972

[B5] Bashashati A. Fatourechi M. Ward R. K. Birch G. E. (2007). A survey of signal processing algorithms in brain–computer interfaces based on electrical brain signals. J. Neural Eng. 4, R32–R57. 10.1088/1741-2560/4/2/R03 17409474

[B6] Bashashati H. Ward R. K. Birch G. E. Bashashati A. (2015). Comparing different classifiers in sensory motor brain computer interfaces. PLoS ONE 10:e0129435. 10.1371/journal.pone.0129435 26090799 PMC4474725

[B7] Bell A. J. Sejnowski T. J. (1995). An information-maximization approach to blind separation and blind deconvolution. Neural Comput. 7, 1129–1159. 10.1162/neco.1995.7.6.1129 7584893

[B8] Belouchrani A. Abed-Meraim K. Cardoso J.-F. Moulines E. (1997). A blind source separation technique using second-order statistics. IEEE Transac. Signal Proc. 45, 434–444. 10.1109/78.554307

[B9] Bingham E. Hyvärinen A. (2000). A fast fixed-point algorithm for independent component analysis of complex valued signals. Int. J. Neural Syst. 10, 1–8. 10.1142/S0129065700000028 10798706

[B10] Bobrov P. Frolov A. Husek D. Snasel V. (2014). Clustering the sources of EEG activity during motor imagery by Attractor Neural Network with Increasing Activity (ANNIA). Proc. Fifth Int. Conf. Innov. Bio Inspir. Comput. Appl. 303, 183–191. 10.1007/978-3-319-08156-4_19

[B11] Bobrov P. Isaev M. Korshakov A. Oganesyan V. Kerechanin J. Popodko A.. (2016). Sources of electrophysiological and foci of hemodynamic brain activity most relevant for controlling a hybrid brain–computer interface based on classification of EEG patterns and near-infrared spectrography signals during motor imagery. Hum. Physiol. 42, 241–251. 10.1134/S036211971603004X 29446587

[B12] Cichocki A. Amari S.-I. (2002). Adaptive Blind Signal and Image Processing: Learning Algorithms and Applications. Chichester: John Wiley and Sons.

[B13] Delfosse N. Loubaton P. (1995). Adaptive blind separation of independent sources: a deflation approach. Signal Proc. 45, 59–83. 10.1016/0165-1684(95)00042-C

[B14] Delorme A. Makeig S. (2004). EEGLAB: an open source toolbox for analysis of single-trial EEG dynamics including independent component analysis. J. Neurosci. Methods 134, 9–21. 10.1016/j.jneumeth.2003.10.009 15102499

[B15] Delorme A. Palmer J. Onton J. Oostenveld R. Makeig S. (2012). Independent EEG sources are dipolar. PLoS ONE 7:e30135. 10.1371/journal.pone.0030135 22355308 PMC3280242

[B16] Dharmaprani D. Nguyen H. K. Lewis T. W. Delosangeles D. Willoughby J. O. Pope K. J. (2016). A comparison of independent component analysis algorithms and measures to discriminate between EEG and artifact components, in 2016 38th Annual International Conference of the IEEE Engineering in Medicine and Biology Society (EMBC) (Orlando, FL: IEEE), 825–828. 10.1109/EMBC.2016.7590828 28268452

[B17] Fonov V. Evans A. C. Botteron K. Almli C. R. McKinstry R. C. Collins D. L.. (2011). Unbiased average age-appropriate atlases for pediatric studies. Neuroimage 54, 313–327. 10.1016/j.neuroimage.2010.07.033 20656036 PMC2962759

[B18] Fonov V. S. Evans A. C. McKinstry R. C. Almli C. R. Collins D. L. (2009). Unbiased nonlinear average age-appropriate brain templates from birth to adulthood. Neuroimage 47:S102 10.1016/S1053-8119(09)70884-5

[B19] Frolov A. Aziatskaya G. Bobrov P. Luykmanov R. K. Fedotova I. Húsek D. (2017a). Electrophysiological brain activity during the control of a motor imagery-based brain–computer interface. Hum. Physiol. 43, 501–511. 10.1134/S036211971705005X

[B20] Frolov A. Husek D. Bobrov P. Korshakov A. Chernikova L. Konovalov R. (2012). Sources of EEG activity most relevant to performance of brain-computer interface based on motor imagery. Neural Netw. World 22, 21–37. 10.14311/NNW.2012.22.002

[B21] Frolov A. A. Husek D. Muraviev I. P. Polyakov P. Y. (2007). Boolean factor analysis by attractor neural network. IEEE Trans. Neural Netw. 18, 698–707. 10.1109/TNN.2007.891664 17526337

[B22] Frolov A. A. Mokienko O. Lyukmanov R. Biryukova E. Kotov S. Turbina L.. (2017b). Post-stroke rehabilitation training with a motor-imagery-based brain-computer interface (BCI)-controlled hand exoskeleton: a randomized controlled multicenter trial. Front. Neurosci. 11:400. 10.3389/fnins.2017.00400 28775677 PMC5517482

[B23] Guillot A. Di Rienzo F. Collet C. (2014). The neurofunctional architecture of motor imagery, in Advanced Brain Neuroimaging Topics in Health and Disease-Methods and Applications, eds Papageorgiou T. D. Christopoulos G. I. Smirnakis S. M. (Rijeka: Intech), 433–456.

[B24] Hétu S. Grégoire M. Saimpont A. Coll M.-P. Eugène F. Michon P.-E.. (2013). The neural network of motor imagery: an ALE meta-analysis. Neurosci. Biobehav. Rev. 37, 930–949. 10.1016/j.neubiorev.2013.03.017 23583615

[B25] Höller Y. Bergmann J. Thomschewski A. Kronbichler M. Höller P. Crone J. S.. (2013). Comparison of EEG-features and classification methods for motor imagery in patients with disorders of consciousness. PloS ONE 8:e80479. 10.1371/journal.pone.0080479 24282545 PMC3839976

[B26] Hyvarinen A. (2001). Blind source separation by nonstationarity of variance: a cumulant-based approach. IEEE Transac. Neural Netw. 12, 1471–1474. 10.1109/72.963782 18249975

[B27] Hyvärinen A. Karhunen J. Oja E. (2004). Independent Component Analysis. New York, NY: John Wiley and Sons.

[B28] Kachenoura A. Albera L. Senhadji L. Comon P. (2007). ICA: a potential tool for BCI systems. IEEE Signal Process. Mag. 25, 57–68. 10.1109/MSP.2008.4408442

[B29] Kobler R. Hirata M. Hashimoto H. Dowaki R. Sburlea A. I. Müller-Putz G. (2019). Simultaneous decoding of velocity and speed during executed and observed tracking movements: an MEG study, in Proceedings of the 8th Graz Brain-Computer Interface Conference 2019: Bridging Science and Application. Graz: Verlag der Technischen Universität Graz 100–105.

[B30] Lee F. Scherer R. Leeb R. Neuper C. Bischof H. Pfurtscheller G. (2005). A comparative analysis of multi-class EEG classification for brain computer interface, in Proceedings of the 10th Computer Vision Winter Workshop (Graz), 195–204.

[B31] Lee T.-W. Girolami M. Sejnowski T. J. (1999). Independent component analysis using an extended infomax algorithm for mixed subgaussian and supergaussian sources. Neural Comput. 11, 417–441. 10.1162/089976699300016719 9950738

[B32] Lotte F. Bougrain L. Cichocki A. Clerc M. Congedo M. Rakotomamonjy A.. (2018). A review of classification algorithms for EEG-based brain–computer interfaces: a 10 year update. J. Neural Eng. 15:031005. 10.1088/1741-2552/aab2f2 29488902

[B33] Mane R. Chew E. Phua K. S. Ang K. K. Robinson N. Vinod A.. (2019). Prognostic and monitory EEG-biomarkers for BCI upper-limb stroke rehabilitation. IEEE Transac. Neural Syst. Rehabil. Eng. 27, 1654–1664. 10.1109/TNSRE.2019.2924742 31247558

[B34] Nam C. S. Jeon Y. Kim Y.-J. Lee I. Park K. (2011). Movement imagery-related lateralization of event-related (de) synchronization (ERD/ERS): motor-imagery duration effects. Clin. Neurophysiol. 122, 567–577. 10.1016/j.clinph.2010.08.002 20800538

[B35] Niedermeyer E. Da Silva F. L. (2005). Electroencephalography: Basic Principles, Clinical Applications, and Related Fields. Philadelphia, PA: Lippincott Williams and Wilkins.

[B36] Ono T. Shindo K. Kawashima K. Ota N. Ito M. Ota T.. (2014). Brain-computer interface with somatosensory feedback improves functional recovery from severe hemiplegia due to chronic stroke. Front. Neuroeng. 7:19. 10.3389/fneng.2014.00019 25071543 PMC4083225

[B37] Palmer J. A. Kreutz-Delgado K. Makeig S. (2006). Super-Gaussian mixture source model for ICA, in International Conference on Independent Component Analysis and Signal Separation (Charleston, SC: Springer), 854–861.

[B38] Palmer J. A. Kreutz-Delgado K. Makeig S. (2012). AMICA: An Adaptive Mixture of Independent Component Analyzers With Shared Components. Swartz Center for Computatonal Neursoscience, University of California San Diego, Technical Report.

[B39] Palmer J. A. Makeig S. Kreutz-Delgado K. Rao B. D. (2008). Newton method for the ICA mixture model, in Acoustics, Speech and Signal Processing. 2008. ICASSP 2008 (IEEE International Conference on: IEEE) (Las Vegas, NV), 1805–1808.

[B40] Ramoser H. Muller-Gerking J. Pfurtscheller G. (2000). Optimal spatial filtering of single trial EEG during imagined hand movement. IEEE Transa. Rehabil. Eng. 8, 441–446. 10.1109/86.895946 11204034

[B41] Ramos-Murguialday A. Broetz D. Rea M. Laer L. Yilmaz O. Brasil F. L.. (2013). Brain-machine interface in chronic stroke rehabilitation: a controlled study. Ann. Neurol. 74, 100–108. 10.1002/ana.23879 23494615 PMC3700597

[B42] Rizzolatti G. Cattaneo L. Fabbri-Destro M. Rozzi S. (2014). Cortical mechanisms underlying the organization of goal-directed actions and mirror neuron-based action understanding. Physiol. Rev. 94, 655–706. 10.1152/physrev.00009.2013 24692357

[B43] Sarin M. Verma A. Mehta D. H. Shukla P. K. Verma S. (2020). Automated ocular artifacts identification and removal from eeg data using hybrid machine learning methods, in 2020 7th International Conference on Signal Processing and Integrated Networks (SPIN) (Noida: IEEE), 1054–1059.

[B44] Tadel F. Baillet S. Mosher J. C. Pantazis D. Leahy R. M. (2011). Brainstorm: a user-friendly application for MEG/EEG analysis. Comput. Intell. Neurosci. 2011:879716. 10.1155/2011/879716 21584256 PMC3090754

[B45] Vasilyev A. Liburkina S. Kaplan A. Y. (2016). Lateralization of EEG patterns in humans during motor imagery of arm movements in the brain-computer interface. Zhurnal Vysshei Nervnoi Deiatelnosti Imeni I.P. Pavlova. 66, 302–312. 30695412

[B46] Vieira S. M. Kaymak U. Sousa J. M. (2010). Cohen's kappa coefficient as a performance measure for feature selection, in International Conference on Fuzzy Systems (Barcelona: IEEE), 1–8.

[B47] Xiaopei W. Zhou B. Lv Z. Zhang C. (2019). To explore the potentials of independent component analysis in brain-computer interface of motor imagery. IEEE J. Biomed. Health Inform. 24, 775–787. 10.1109/JBHI.2019.2922976 31217132

[B48] Zhou B. Wu X. Lv Z. Zhang L. Guo X. (2016). A fully automated trial selection method for optimization of motor imagery based brain-computer interface. PloS ONE 11:e0162657. 10.1371/journal.pone.0162657 27631789 PMC5025076

[B49] Ziehe A. Laskov P. Nolte G. MãŽller K.-R. (2004). A fast algorithm for joint diagonalization with non-orthogonal transformations and its application to blind source separation. J. Mach. Learn. Res. 5, 777–800. Available online at: http://www.jmlr.org/papers/v5/ziehe04a.html

